# A biophoton method for identifying the quality states of fresh Chinese herbs

**DOI:** 10.3389/fphar.2023.1140117

**Published:** 2023-03-20

**Authors:** Baorui Cao, Zhiying Wang, Jiayi Zhang, Jialei Fu, Zhongwen Zhang, Jinxin Du, Tingting Deng, Jingxiang Pang, Meina Yang, Jinxiang Han

**Affiliations:** ^1^ Biomedical Sciences College and Shandong Medicinal Biotechnology Centre, First Affiliated Hospital of Shandong First Medical University, Shandong First Medical University and Shandong Academy of Medical Sciences, Jinan, China; ^2^ NHC Key Laboratory of Biotechnology Drugs, Shandong Academy of Medical Sciences, Jinan, China; ^3^ Shandong Academy of Chinese Medicine, Jinan, China; ^4^ Department of Endocrinology, The First Affiliated Hospital of Shandong First Medical University, Jinan, China; ^5^ Shandong University of Traditional Chinese Medicine, Jinan, China

**Keywords:** biophoton, quality state, fresh Chinese herb, motherwort, safflower, UPLC, characteristic parameter

## Abstract

**Introduction:** The quality of Chinese herbs is the basis for ensuring their safety and efficacy. However, the quality evaluation system is imperfect. In particular, there is a lack of quality evaluation methods for fresh Chinese herbs during growth. The biophoton is a common phenomenon and provides complete information about the interior of the living system, which is consistent with the holistic concept of traditional Chinese medicine. Therefore, we aim to correlate the biophoton characteristics with the quality states to find the biophoton parameters that can characterize the quality states of fresh Chinese herbs.

**Methods:** The biophoton characteristics of motherwort and safflower were measured and characterized by the counts per second (CPS) in the steady state and the initial intensity (I_0_) and coherent time (T) of delayed luminescence. The active ingredient content was measured by ultra-high-performance liquid chromatography (UPLC). The pigment content of motherwort leaves was measured by UV spectrophotometry. The t-test and correlation analysis were performed on the experimental results.

**Results:** The CPS and I_0_ of motherwort and I_0_ of safflower showed a significant downward trend during the growth process, and their active ingredient content showed a trend that increased and then decreased. The CPS, I_0_, and the content of active ingredients and pigments in a healthy state were significantly higher than those in a poor state, while T showed the opposite results. The CPS and I_0_ were all significantly and positively correlated with the content of active ingredients and pigments, while the T of motherwort showed the opposite results.

**Conclusion:** It is feasible to identify the quality states of fresh Chinese herbs by using their biophoton characteristics. Both CPS and I_0_ have better correlations with the quality states and can be considered characteristic parameters of the quality of fresh Chinese herbs.

## 1 Introduction

The quality of Chinese herbs is the key to ensuring their stability, safety, and efficacy (Wu et al., 2018; Li et al., 2022). The traditional quality evaluation methods are mainly based on the “shape,” “color,” “qi,” “taste,” and other characteristics of Chinese herbs to determine their authenticity and quality (Chen et al., 2021). Some scholars have proposed the idea of “quality evaluation by morphological identification” (Xie, 1994). The current quality evaluation methods of Chinese herbs rely more on chromatographic pharmacodynamics (Ma et al., 2022; Yan and Zou, 2022), serum pharmacochemistry (Wu et al., 2019; Tang et al., 2022), pharmacokinetics (Ouyang et al., 2022; Shang et al., 2022), metabonomics (Ning et al., 2013; Feng et al., 2020), quality markers (Zhang et al., 2018a; Ren et al., 2020), biological effect detection (Wang et al., 2013; Zhang et al., 2022), and other modern scientific techniques, while the most common method relies on the fingerprinting techniques to determine the content of active ingredients to evaluate and control the quality of Chinese herbs (Zhang and Jiang, 2021). A holistic view and evidence-based treatment are the significant features of the theoretical system of Chinese herbs, and the quality of Chinese herbs is the result of the overall performance of its multi-components and multi-effects (Liu et al., 2019). However, the components of Chinese herbs are extremely complex, so it is difficult to characterize them completely, and the detection indicators are too singular (Zhu et al., 2017; Lin et al., 2020). Due to the multi-component, multi-target, and multi-pathway characteristics of Chinese herbs, the basic research on pharmacodynamic substances is relatively weak (Wang et al., 2013; Zhang et al., 2018b). Quality control focuses more on small molecular components, but less on macromolecules, such as sugars, proteins, amino acids, and peptides (Wang et al., 2021a). The quality evaluation of Chinese herbs often refers to the detection methods of Western medicine and chemical medicine, but they are not completely applicable to Chinese herbs. Moreover, the cultivation of Chinese herbs is separated from the quality control system. The natural environment, cultivation techniques, harvesting period, and processing methods are all important factors affecting the quality. Therefore, there is a lack of quality evaluation methods in the growth process of fresh Chinese herbs.

Motherwort is the fresh or dried aerial part of *Leonurus japonicus* Houtt., a plant of the Labiatae family. It tastes bitter and pungent, has a slightly cold nature, and is associated with the liver, pericardium, and bladder meridians. It has the effects of promoting blood circulation, regulating menstruation, promoting urination, dispersing swellings, clearing heat, and resolving toxins (Commission, 2020). It is commonly used in the clinical treatment of common gynecological diseases. Relevant studies have shown that motherwort also has significant therapeutic effects on cardiovascular disease (Rong et al., 2022). Safflower is the dried flower of the Compositae plant *Carthamus tinctorius* L. It tastes pungent, has a warm nature, and is associated with the heart and liver meridians. It has the effects of promoting blood circulation, regulating meridians, dispersing blood stasis, and relieving pain (Commission, 2020). It is widely used in the clinical treatment of anemia, coronary heart disease, angina pectoris, stroke, and other diseases (Wang et al., 2019; Wang et al., 2021b).

Biophoton emission is a common phenomenon in living organisms. Its intensity is very low. It is closely related to the basic life processes in the organisms, such as cell division, information transmission, photosynthesis, and metabolism, and is extremely sensitive to slight changes in the external environment (Popp, 2003; Cifra and Pospisil, 2014). Biophoton emission is believed to be generated in the process of the mutual transition between the high-energy and low-energy states of biological molecules and carries complete information about the interior of living systems (Popp et al., 1988; Gu, 2012). Some scholars believe that the holistic concept of traditional Chinese medicine (TCM) and the biophotonic coherence theory are commensurable and consistent in some aspects (Yang and Han, 2013; Zhao and Han, 2013; Fan et al., 2017). Therefore, the coherence theory of biophoton radiation was integrated with the holistic concept of TCM, and thus the concept of “quantum TCM ” was proposed.

Our research group used honeysuckle buds and *Platycodon grandiflorum* leaves as the subjects of a preliminary study, found that the biophoton characteristics of fresh Chinese herbs at different growth stages, in different growth states, and of different species differed significantly, and revealed three characteristic parameters, namely, counts per second (CPS) of spontaneous photon emission (SPE) in the steady state, and initial intensity (I_0_) and coherent time (T) of delayed luminescence (DL). Therefore, in this study, combined with the previous experimental methods, two herbs with different medicinal parts were selected to analyze the differences in biophoton characteristics between different samples and to verify whether these methods are broadly applicable and reproducible. These results were then compared with the content of the active ingredients to analyze the feasibility of using the biophoton characteristics of fresh Chinese herbs for quality evaluation.

## 2 Materials and methods

### 2.1 Cultivation of Chinese herbs

Motherwort and safflower were selected for cultivation based on their medicinal parts, medicinal properties, growth environment, and other factors.

Before sowing, the soil was turned to a depth of 30 cm and sheep manure was applied as a base fertilizer. The motherwort was sown in 3–5-cm deep holes at a 40-cm row spacing. For sowing safflower, holes were dug 3–5 cm deep with a row spacing of 30 cm and a hole spacing of 25 cm. Irrigation was carried out once every 5 days, and timely drainage was conducted when rainfall was concentrated during the rainy season. Weeding was performed once every 2 weeks during the vigorous growth period. Pesticides were sprayed in a timely manner to prevent the occurrence or spread of pests and diseases.

The motherwort was divided into three groups: the control group (normal irrigation, and normal fertilization; CG), the non-fertilization group (normal irrigation, but no fertilization; NG), and the drought group (normal fertilization, but less irrigation; DG). Each group was planted within 5 m^2^ of the same plot, with an interval of more than 2 m between the two adjacent groups to avoid any mutual influence between the treatment groups. The herbs were fertilized once a month with 200 g of Stanley compound fertilizer. The drought group was irrigated once every 2 weeks, while the other groups were irrigated once every 5 days. The other factors were the same for all groups. The safflower was divided into two groups (the healthy state and the poor state) according to the height of the plant, whether the stems and leaves were strong, whether the leaves and flowers were bright, and whether there were any pests or diseases.

### 2.2 Biophoton measurement

#### 2.2.1 The biophoton system

A YPMS-2 biophoton measurement system (Meluna Research B.V. Germermalsen, the Netherlands) was used in the study. It was mainly composed of a sample darkroom, an excitation light source, a photomultiplier tube, a high-voltage power supply, a photon counting unit, a thermoelectric cooler, and a computer with photon counting data software. The photomultiplier tube (QA9863; ET Enterprises, United Kingdom) was the core component.

#### 2.2.2 Samples and preparation

In this study, motherwort leaves and safflower flowers in different growth stages and different states were used as experimental samples. The growth process of motherwort leaves was divided into five stages. The growth process of safflower included the bud stage and the anthesis stage, each of which was further divided into four stages. Samples were collected in the morning and afternoon to avoid strong midday light exposure at noon. In case of extreme weather conditions, sampling and experiments were halted to ensure the consistency of the natural environment when obtaining samples for experiments. The sampling was random. The collected samples were placed into a bag for fresh-keeping and then immediately stored in an ice box. The samples were cleaned with double distilled water, blotted dry with absorbent papers, and photographed to record the states. The samples were then dark-adapted for 1 h in the instrument darkroom for subsequent measurements. The time interval between the sample collection and dark adaptation was consistent for each experiment (20 min).

#### 2.2.3 Measurement methods

The room temperature was set at 20°C, and the instrument was switched on to cool for 2 h before starting the measurement. The background noise of the instrument without the samples was first measured continuously for 10 min. After the dark adaptation, the SPE of the samples was measured continuously for 10 min. The samples were then irradiated with a white LED for 15 s to detect their delayed DL and measured continuously for 5 min, and the experiment was repeated three times for each sample. After the samples were removed, the background noise of the instrument was measured again continuously for another 10 min. The weight of each sample was recorded immediately after the measurement using an electronic balance, and the thickness of the motherwort leaves was measured using a leaf thickness gauge. Throughout the entire experiment, the measurement time interval was 1 s, and the height of the sample table in the darkroom, i.e., the distance between the sample and the light source, was always kept constant.

#### 2.2.4 Data processing

We measured the biophoton intensity of motherwort and safflower. To find the parameters that represented the biophoton characteristics of Chinese herbs and then to quantitatively compare the differences in the biophoton characteristics in different groups, we initially characterized the SPE properties with CPS and characterized the DL properties with I_0_ and T. The original data of motherwort were normalized using sample weight and thickness, while the safflower data were normalized by weight only (the flower of safflower is nearly spherical, the weight (volume) is only related to the radius, and the surface area is only related to the radius; therefore, it only needs to be normalized by weight instead of surface area). The equations for calculating these parameters are as follows:

CPS=N−n (1)

I0=A×csch⁡^2C, (2)

T=B×lnm×sinh⁡C+m×sinh⁡^2C+1−C. (3)

Eq. 1 is the calculation formula of CPS; N is the average number of photons per second during the sample measurement; n is the average number of photons per second of the background noise without any samples. Eq. 2 and Eq. 3 are the calculation formulas for the two parameters of DL; A, B, and C are the three parameters in the Gu function (Gu, 2012); A is related to the nature of the sample, the structure of the detection system, and lighting conditions; B is a time parameter that is only related to the nature of the sample; C is a characteristic parameter related to the initial luminous intensity of the sample; m is a characteristic factor and considered equal to 3 in this study.

### 2.3 Measurement of the active ingredient content

#### 2.3.1 Samples and preparation

After the biophoton detection, the samples from different groups at the same stage were collected and washed with double distilled water, placed into an oven to dry for 24 h (the safflower was dried at 45°C, and the motherwort was dried at 60°C), and ground into a powder with a multi-function grinder. The powder was then sieved through a No. 3 Chinese medicine sieve (50-mesh), and placed in a 50-mL centrifuge tube. Finally, the processed samples were labeled and stored in a cool, dry, and ventilated place.

For the extraction of leonurine hydrochloride, 0.5 g of motherwort powder was accurately weighed and placed in a conical flask with a stopper. Then, 25 mL of 70% ethanol was accurately added to the flask, and the total weight of the mixture was weighed. The mixture was heated and refluxed for 2 h. After cooling, it was reweighed and the lost weight was replaced with 70% ethanol. Finally, the sample was filtered through a 0.22-μm membrane.

For the extraction of hydroxy safflower yellow A (HSYA) and kaempferol, 0.5 g of safflower powder was accurately weighed and placed in a conical flask with a stopper. Then, 25 mL of methanol was accurately added to the flask, and the mixture was weighed. A PL-S40 digital display ultrasonic cleaner (Dongguan Kangshijie Ultrasonic Technology Co., Ltd., China) was used for ultrasonic treatment for 40 min. After cooling, the mixture was reweighed and the lost weight was replaced with methanol. Finally, the sample was filtered through a 0.22-μm membrane.

#### 2.3.2 Ultra-high-performance liquid chromatography analysis

Ultra-high-performance liquid chromatography (UPLC) analysis was performed on a Waters ultra-high-performance liquid chromatograph (Waters, Milford, MA, USA), using an ACQUITY UPLC BEH C18 chromatographic column (50 × 2.1, 1.7 μm). The column temperature was 25°C. Leonurine hydrochloride was determined by using a mobile phase of acetonitrile and a 0.1% phosphoric acid solution of 0.4% sodium octane sulfonate (35:65, v/v). The injection volume was 10 μL. The flow rate was 1.0 mL/min. The detection wavelength was 277 nm. HSYA and kaempferol were detected by using methanol (A) and a 0.75% (v/v) phosphoric acid solution (B) as the mobile phase, and eluted in a gradient according to the following procedure: 35% (A) from 0 to 5 min, 40% (A) from 5 to 10 min, 45% (A) from 10 to 20 min, 55% (A) from 30 to 35 min, and 40% (A) from 35 to 45 min. The injection volume was 10 μL. The flow rate was 0.5 mL/min. The detection wavelength was 360 nm. A standard solution of leonurine hydrochloride containing 0.5 mg of leonurine hydrochloride per 1 mL was prepared with 70% ethanol, and a standard solution containing 0.13 mg of HSYA and 9 μg of kaempferol per 1 mL was prepared with methanol. The methods were shown to have good precision, repeatability, and stability for each chemical component, and a linear regression equation was obtained for each component.

### 2.4 Measurement of the pigment content

Samples of fresh motherwort leaf from the same parts were randomly selected, cleaned with double-distilled water, blotted dry with absorbent papers, and sampled by punching holes while avoiding the main veins. A 50-mL centrifuge tube was wrapped in aluminum foil, and 0.1000 g of sample and 15 mL of extraction solution (alcohol and acetone mixed 1:1 by volume) were added. The mixture was incubated in the dark for 24 h until the leaves had completely discolored. During this period, the samples were shaken and mixed at least twice.

After 24 h, the absorbance of the extract was measured at 474, 642, and 665 nm using an ultraviolet-visible spectrophotometer with the mixed extract as a blank control. The following formulae were used to calculate the concentration and content of each pigment (Huang et al., 2013):

Ca=9.99×A665 nm−0.0872×A642 nm, (4)

Cb=17.7×A642 nm−3.04×A665 nm, (5)

$$C\left(a+b\right)=Ca+Cb,$$ (6)

Cc=4.92×A474nm−0.0255×Ca−0.225×Cb, (7)

$$M=C\times \frac{V}{g}.$$ (8)

Eqs 4–7 are the formulas for calculating the concentration of the pigments; Ca, Cb, C (a+b), and Cc respectively represent the concentration (mg/L) of chlorophyll a, chlorophyll b, total chlorophyll, and total carotenoids; M is the content of the pigment (mg/g); V is the total volume (L) of the extract; g is the weight (g) of the sample.

### 2.5 Data analysis

GraphPad Prism 9.0.0 software (San Diego, California, United States, www.graphpad.com) was used for the statistical analysis of all results. The two-tailed unpaired Student’s t-test was used to show the differences between different groups, and the correlation analysis based on the Pearson correlation coefficient was used to show the correlation between each biophoton parameter and the content of active ingredients and pigments. In all statistical tests, *p* ≤ 0.05 was considered to be significant.

## 3 Results

### 3.1 Analysis of the biophoton characteristics

**FIGURE 1:**
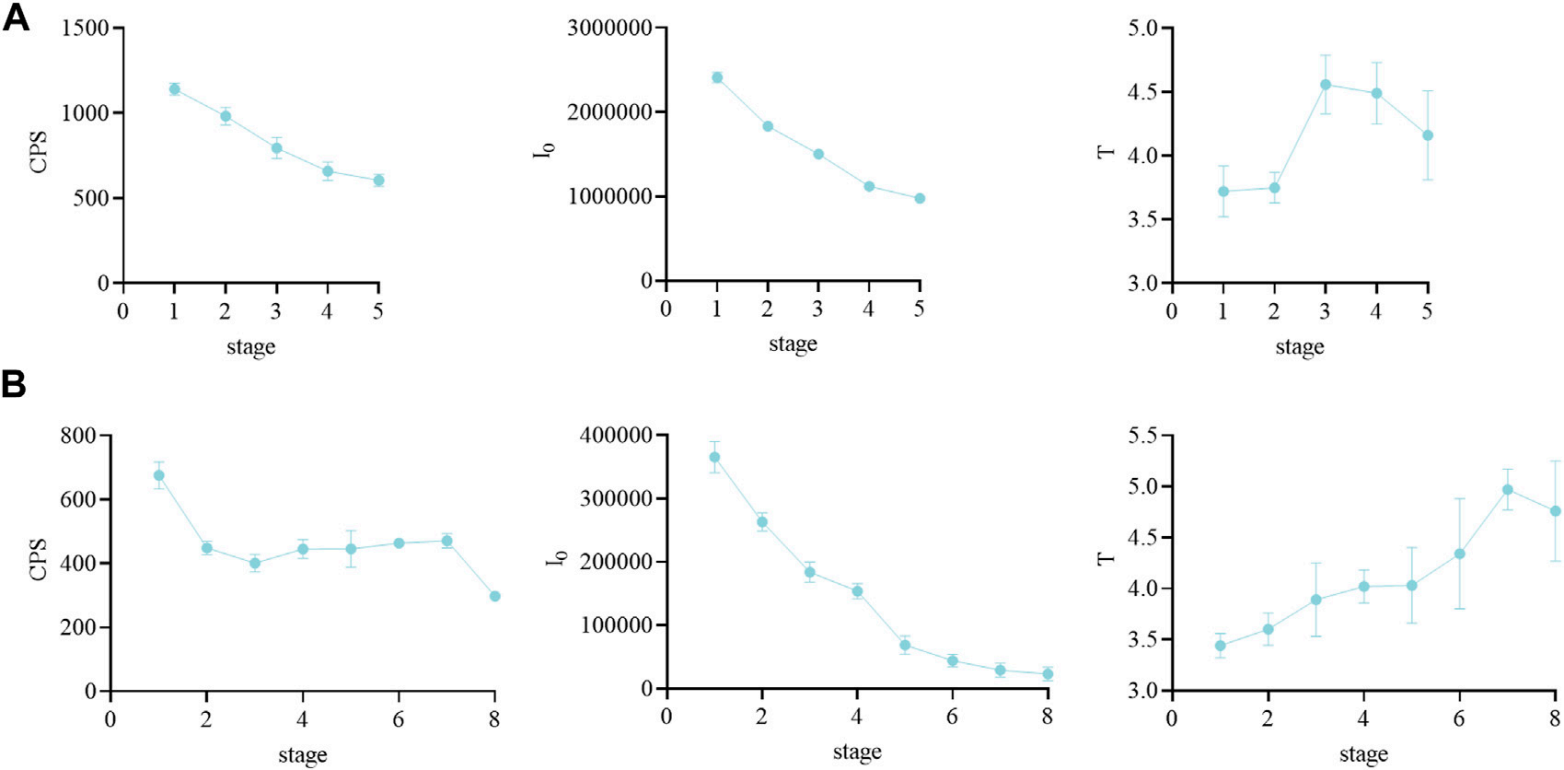
The biophoton characteristics of motherwort and safflower in different growth stages. **(A)** Motherwort. **(B)** Safflower.

**FIGURE 2:**
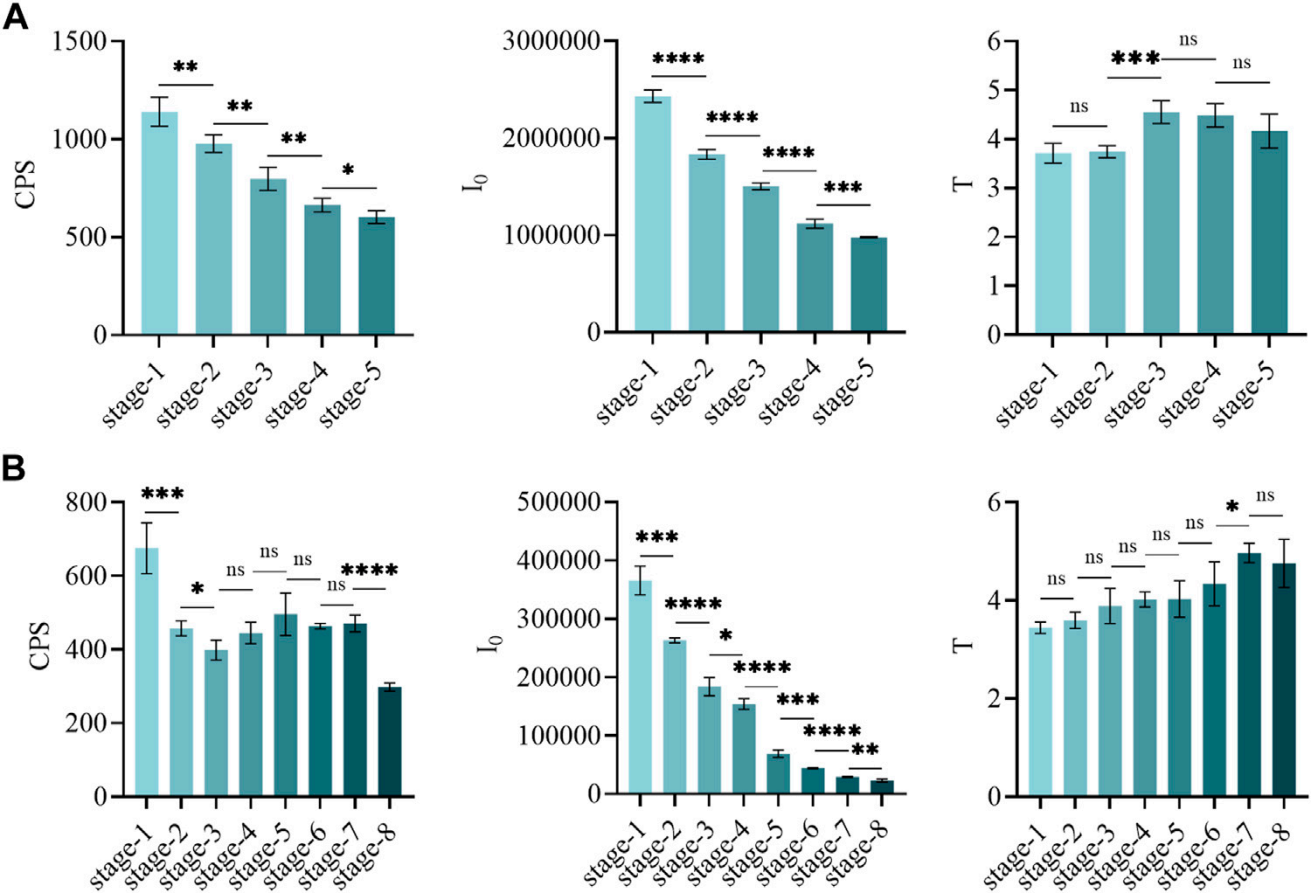
The t-test in biophoton parameters of motherwort and safflower in different growth stages. **(A)** Motherwort. **(B)** Safflower.

During the growth of motherwort and safflower, their biophoton characteristics were visibly changed (Figure 1). Figure 2 displays that the CPS and I_0_ of motherwort and the I_0_ of safflower showed a significant downward trend during their growth process, while the CPS and T of safflower and the T of motherwort showed basically no changes. This shows that the biophoton parameter I_0_ can well distinguish the different growth stages of Chinese herbs. The CPS seems to play a similar role, especially in the growth process of motherwort. However, its applicability needs further experimental verification. The T has no significant difference, and the data is not stable enough, so it cannot be used to distinguish the different growth stages of Chinese herbs.

**FIGURE 3:**
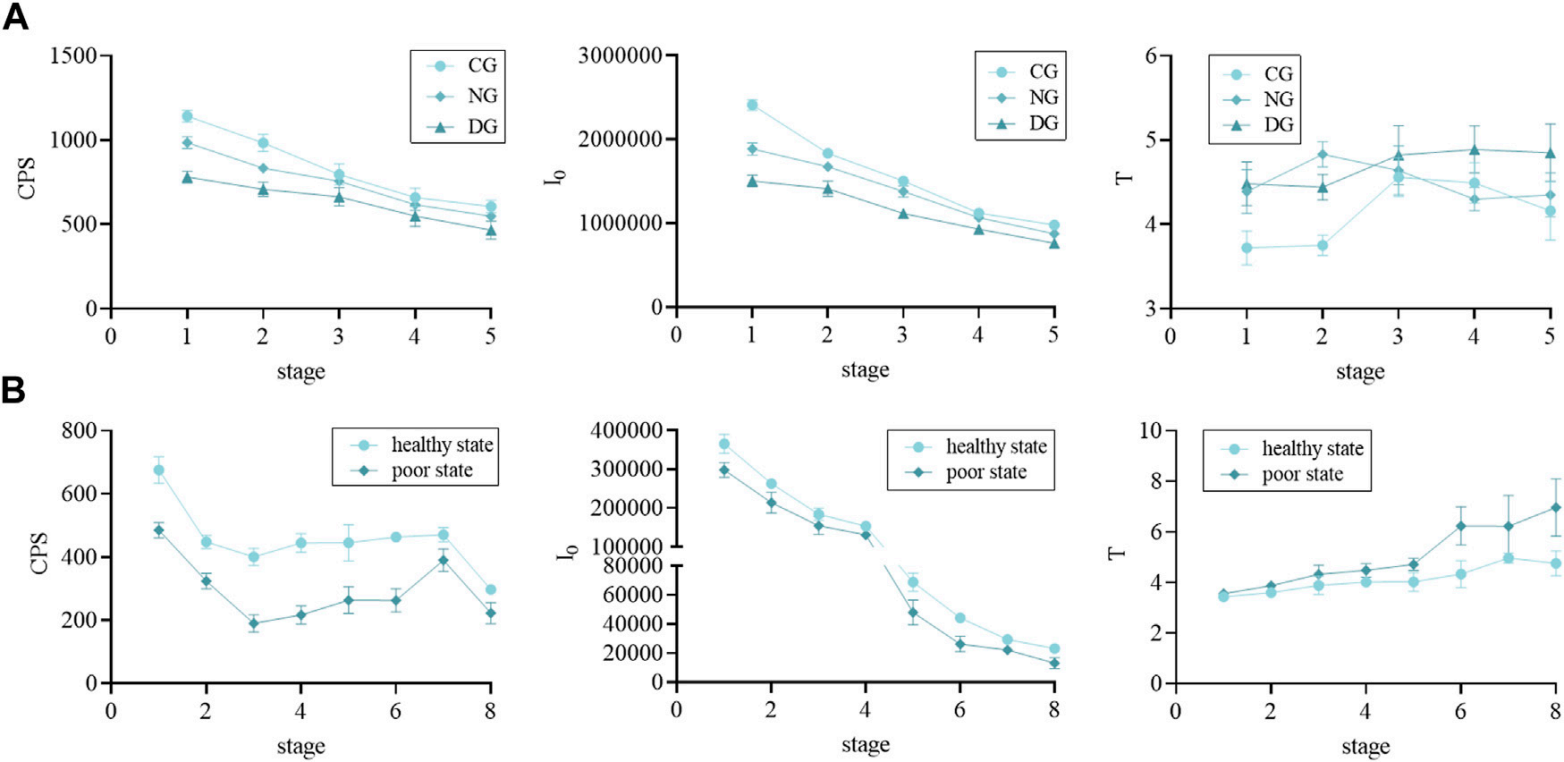
The biophoton characteristics of motherwort and safflower in different growth states. **(A)** Motherwort. **(B)** Safflower.

**FIGURE 4:**
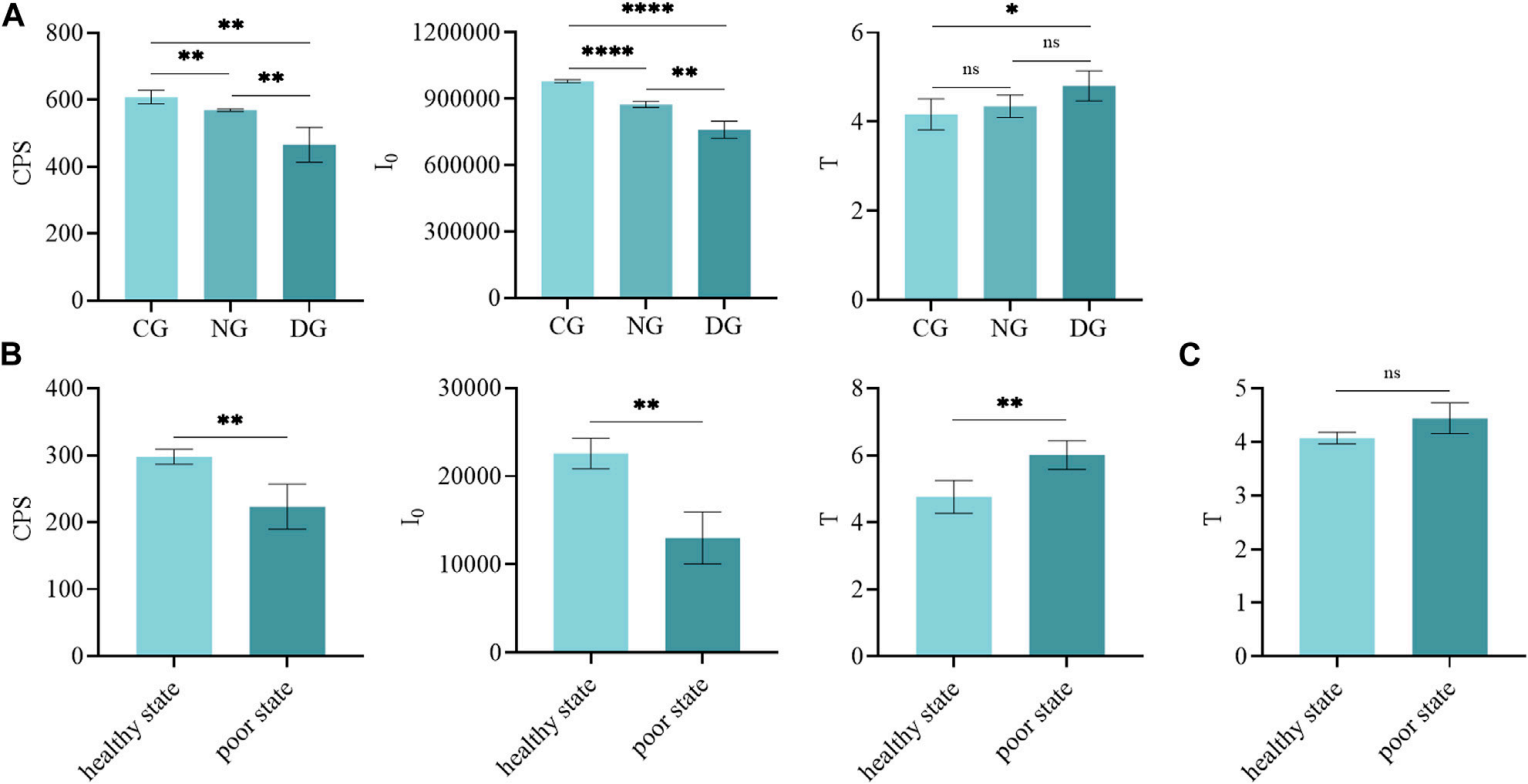
The t-test in biophoton parameters of motherwort and safflower in different growth states. **(A)** Motherwort. **(B)** Safflower. **(C)** Safflower bud.

We found that there were differences in the biophoton characteristics of motherwort and safflower in different groups (Figure 3). Figure 4 illustrates that there were significant differences in both CPS and I_0_ between different groups of motherwort and safflower, and these parameters in a healthy state were significantly higher than those in a poor state. As for the parameter T, only the difference in motherwort between the control group and the drought group was significant, and that of the drought group was significantly higher than that of the control group. The T of safflower in a poor state was significantly higher, however, there was no significant difference in the bud stage. These findings suggest that the CPS of SPE and the I_0_ of DL have a good correlation with the quality states of fresh Chinese herbs, and can be considered as biophoton index parameters for characterizing the quality of fresh Chinese herbs.

### 3.2 Analysis of the active ingredient content based on ultra-high-performance liquid chromatography

**FIGURE 5:**
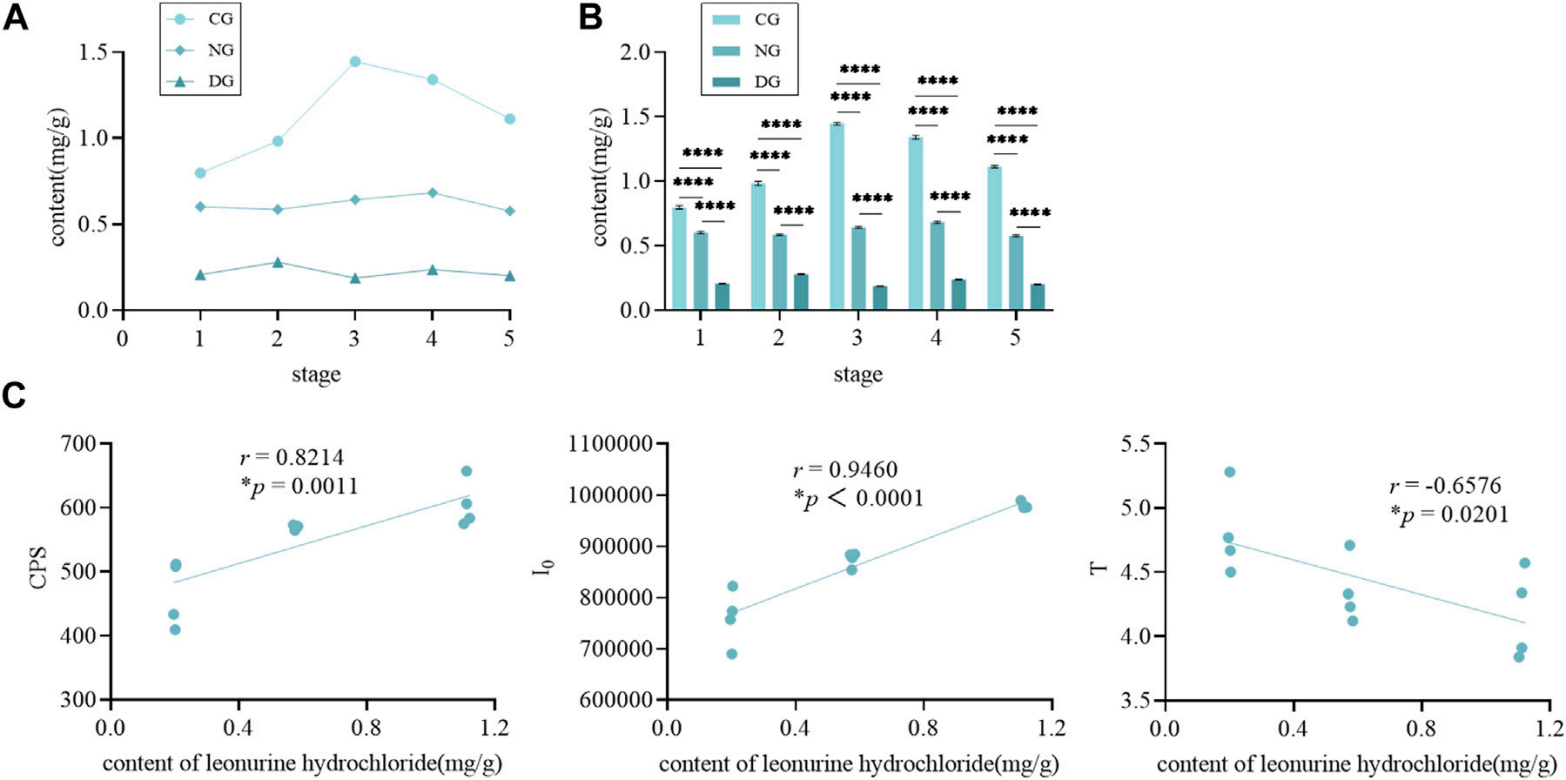
The UPLC and correlation analysis of motherwort. **(A)** The content of leonurine hydrochloride in different groups during the growth of motherwort. **(B)** The t-test of leonurine hydrochloride content in different groups. **(C)** The correlation analysis between leonurine hydrochloride content and biophoton parameters.

Taking the control group of motherwort as an example, the content of leonurine hydrochloride gradually increased in the early growth period, reached the highest level in stage 3 (the measurement date was 30 September 2022, and the growth period was approximately 3 months), and then slightly decreased in the following two stages (Figure 5A). Figure 5B shows that the content of leonurine hydrochloride in the control group was significantly higher than that in the other two groups and that in the non-fertilization group was also significantly higher than that in the drought group. Combined with the biophoton measurement results, we found that there was a certain correlation between them. To specifically analyze the potential correlation, we conducted a correlation analysis based on the Pearson correlation coefficient (Figure 5C). We found that the content of leonurine hydrochloride was significantly and positively correlated with the biophoton parameters CPS and I_0_, but negatively correlated with the T. And the absolute value of the correlation coefficient with I_0_ was the highest, while that with T was the lowest. Therefore, CPS and I_0_ can discriminate Chinese herbs with different quality states, while the I_0_ has a better characterization effect.

**FIGURE 6:**
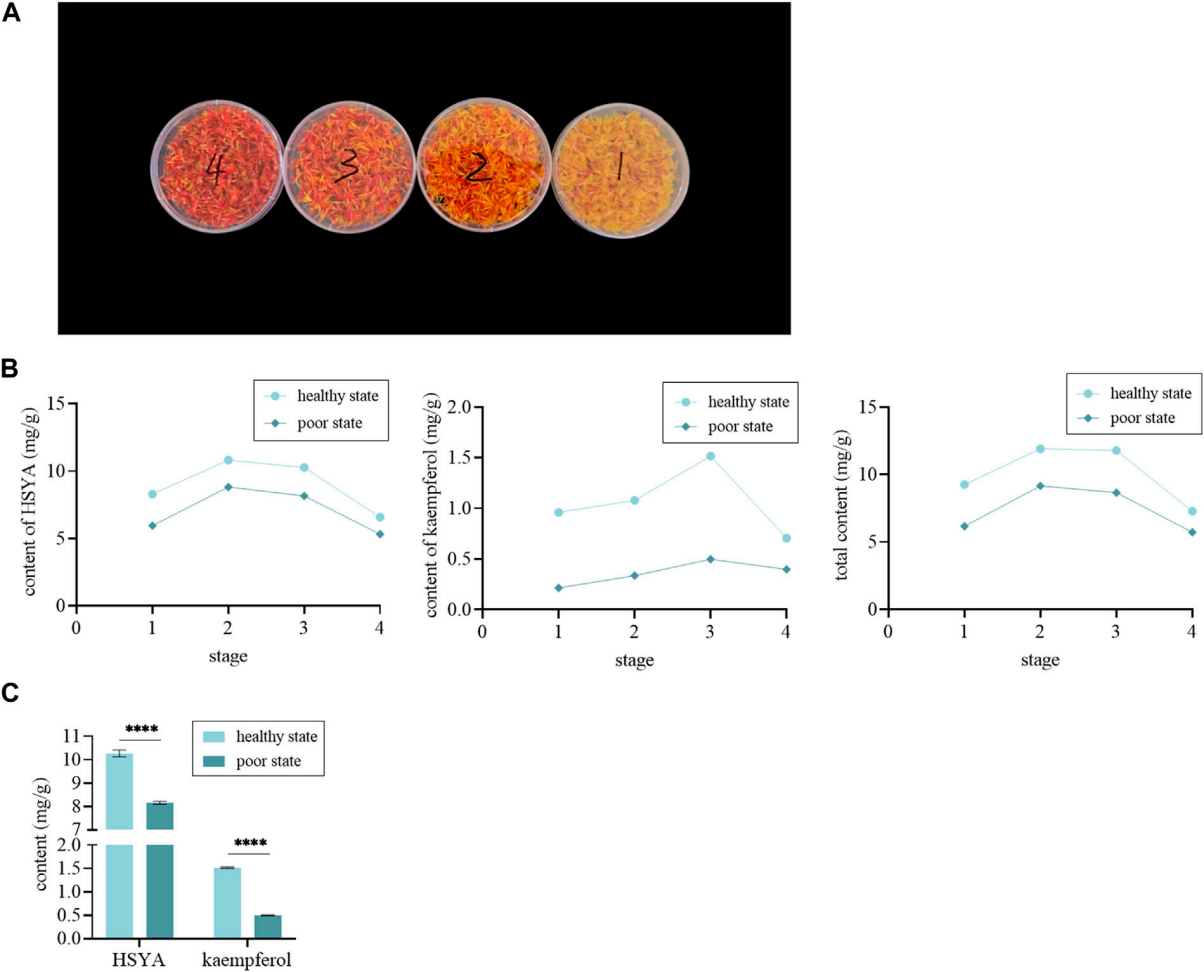
The UPLC analysis of safflower. **(A)** The samples of safflower in different flowering stages. **(B)** The content of HSYA and kaempferol in different states. **(C)** The t-test of HSYA and kaempferol content in different states.

**FIGURE 7:**
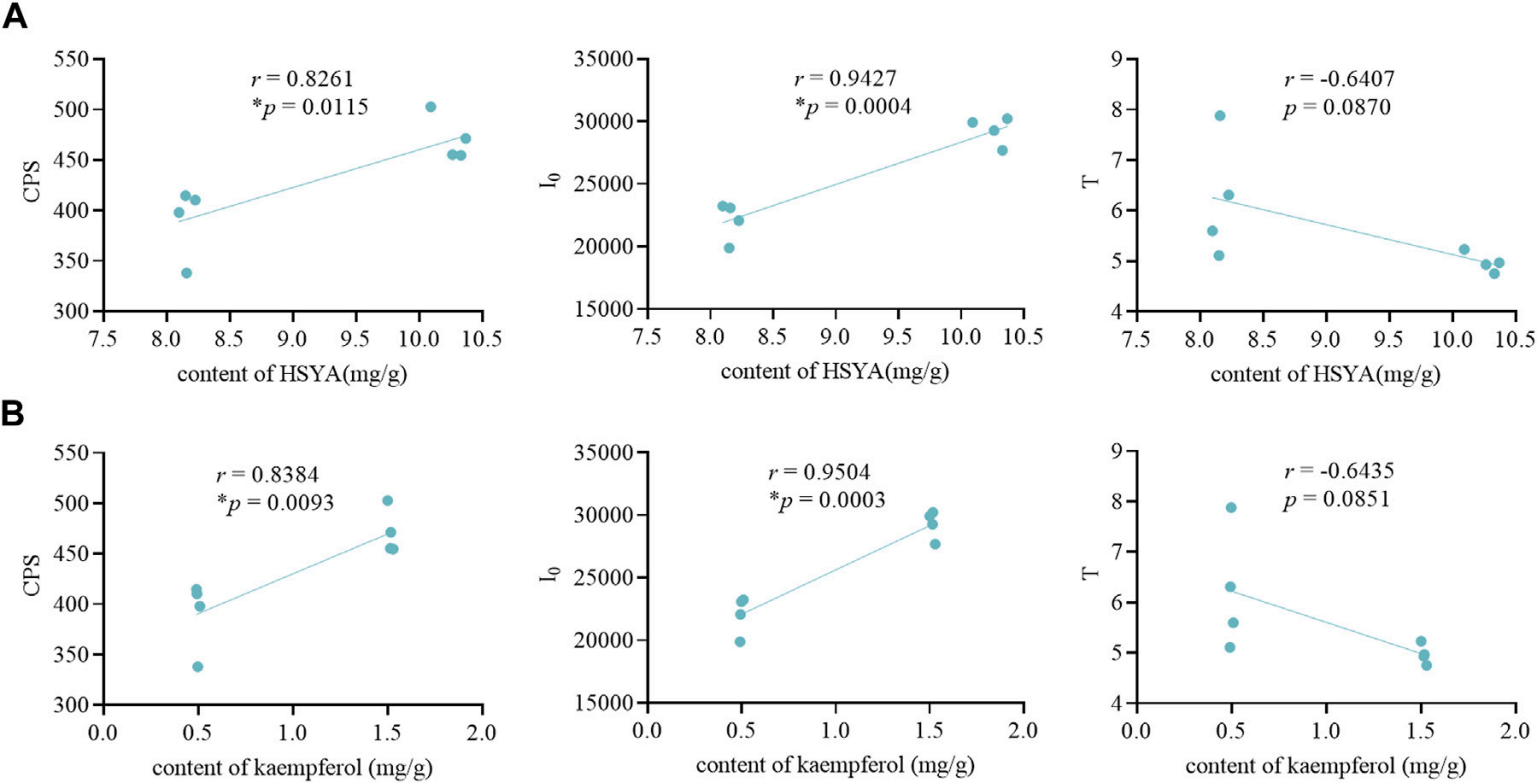
The correlation analysis of safflower. **(A)** The correlation analysis between the content of HSYA and the biophoton parameters. **(B)** The correlation analysis between the content of kaempferol and the biophoton parameters.

The content of HSYA and kaempferol were different in different groups. As shown in Figure 6A, the color of safflower inflorescences in different flowering stages varied from light yellow, dark yellow, and red to dull red. Similarly, the active content also changed (Figure 6B). In stage 2 and stage 3, that was, when the inflorescence changed color from yellow to red, the content of HSYA and kaempferol was relatively high, and met the content criteria (HSYA is 1%, and kaempferol is 0.05%) specified in the Chinese Pharmacopoeia (Commission, 2020). However, the levels in stages 1 and 4 were relatively low and below the content criteria. We found that there were significant differences in the content of active ingredients in different groups, and those in a healthy state were significantly higher (Figure 6C). And the correlation analysis results show that the content of HSYA and kaempferol was significantly and positively correlated with CPS and I_0_, and the correlation with I_0_ was the highest, while there was no significant correlation with T (Figure 7). These resuls are consistent with these of the motherwort.

### 3.3 Analysis of the pigment content in motherwort leaves

**FIGURE 8:**
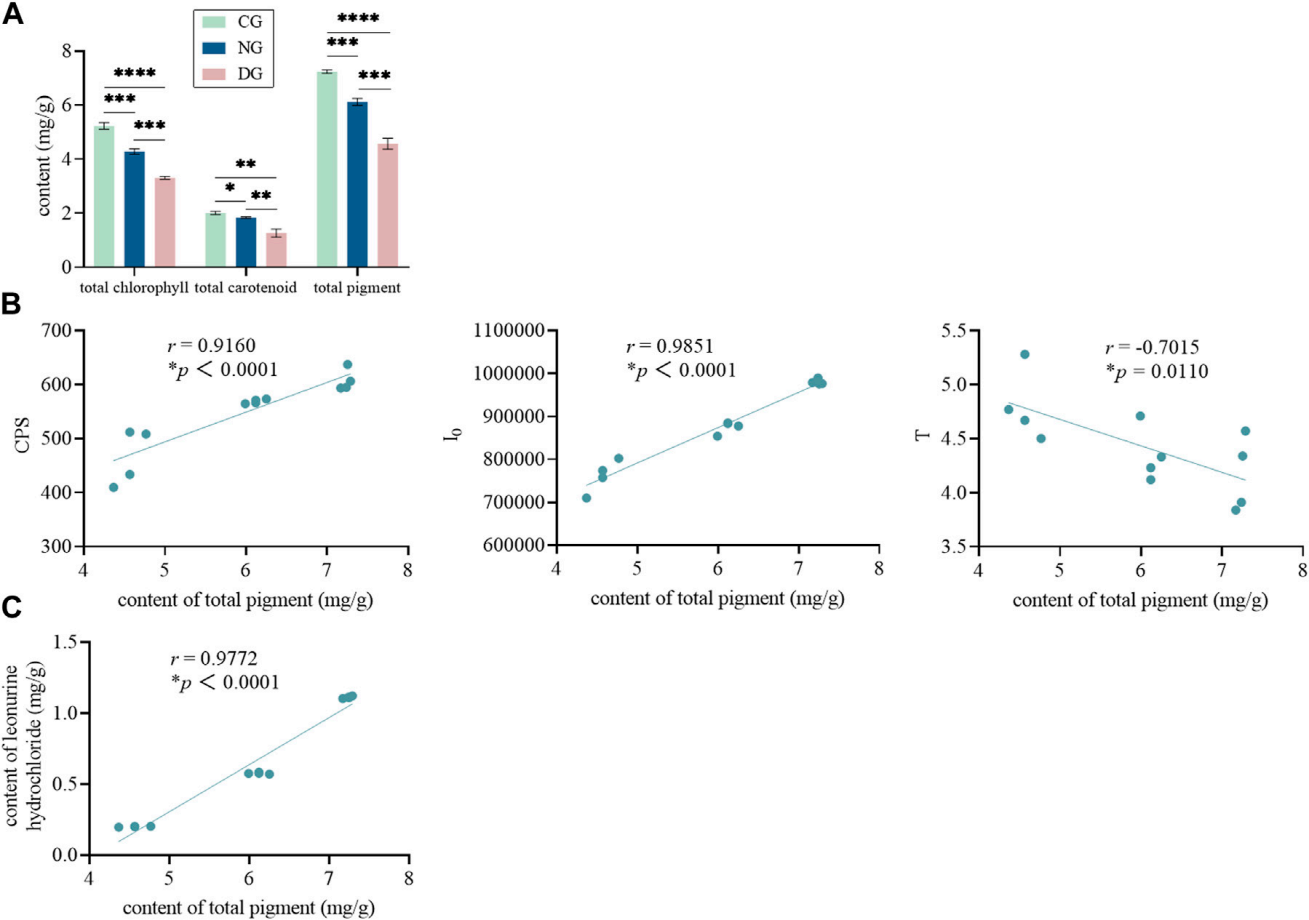
The pigment content and correlation analysis of motherwort leaves in different groups. **(A)** The t-test of the pigment content in different groups. **(B)** The correlation analysis between the pigment content and the biophoton parameters. **(C)** The correlation analysis between the content of pigment and leonurine hydrochloride.

The leaves of green plants contain many pigments, mainly chlorophyll a, chlorophyll b, lutein, and carotenoids. These pigments play an important role in photosynthesis (Cu et al., 2022; Wohlfahrt et al., 2022) and are also an important source of bioluminescence in leaves. We found that the pigment content of motherwort leaves differed significantly between different groups (Figure 8A). The total chlorophyll, total carotenoid, and total pigment contents in the control group were all significantly higher than those in the other two groups, and those in the non-fertilization group were also significantly higher than those in the drought group. The correlation analysis shows that the pigment content of motherwort leaves was significantly correlated with the biophoton characteristics and the content of active ingredients (Figures 8B,C). Among them, the pigment content was significantly and positively correlated with CPS, I_0_, and the content of leonurine hydrochloride, while that with t was significantly but negatively correlated. The results are also consistent with the above results, indicating the good feasibility of the biophoton method for identifying the quality states of fresh Chinese herbs.

## 4 Discussion

The quality of Chinese herbs is the basis for ensuring their safety, efficacy, and stability. However, the current quality evaluation system of Chinese herbs is imperfect and measures and evaluates the quality of Chinese patent medicine after collection and processing. In particular, there is a lack of quality evaluation methods for fresh Chinese herbs during the growth process. The biophoton based on the “coherence theory” reflects the complete information inside an organism as a whole and is very sensitive to weak changes in the external environment, which is consistent with the overall concept of the TCM theory (Fu et al., 2015; Zeng et al., 2016; Fan et al., 2017). Furthermore, the measurement method is simple, fast, and highly sensitive. As a new non-invasive method, it does not damage the measurement samples and has been widely used in medical, food, pharmaceutical, agricultural, environmental health, and other fields (Choi et al., 2016; Kamal and Komatsu, 2016; Murugan et al., 2018; Lukacs et al., 2022). In this study, we used fresh samples of motherwort and safflower as the research objects, explored the relationship between their biophoton characteristics and their quality states, and analyzed the correlation between the biophoton characteristics, active ingredient content, and pigment content, to explore the feasibility and available parameters of using the biophoton meathod to identify the quality of fresh Chinese herbs.

The SPE and DL characteristics of samples are important components of research related to biophoton analysis. The biophoton parameter CPS reflects the SPE of the sample in a steady state. Studies have shown that SPE is related to the active oxygen species produced in the redox metabolic activities in biological systems (Hideg et al., 1990; Popp, 2003). The more vigorous the metabolic activity is, the more biological molecules there are in the excited state, and according to the “coherence theory,” the stronger the coherent radiation is generated. DL is a persistent luminescence phenomenon in biological systems excited by external photons (Xi et al., 2011). The I_0_ reflects the bioluminescence intensity of the sample when the excitation ends, which can be used as a comprehensive indicator of biological metabolic activity. The higher the I0, the higher the overall metabolic activity of the organism. This study found that the CPS and I_0_ of the samples in a healthy state were significantly higher, indicating that there were more excited biomolecules in the samples, the molecular coherence was stronger, and the metabolic activity was more vigorous. The pigment content analysis showed that the pigment content of motherwort leaves in a healthy state was significantly higher. This indicated that under the same external conditions, the samples in a healthy growth state should have stronger photosynthesis and more excited molecules, and therefore, they should have a higher bioluminescence intensity, which was demonstrated in the biophoton detection results.

The coherence time T is a characteristic quantity related only to the properties of the sample itself and characterizes the decay rate of the delayed luminescence kinetic curve of the sample. The better the coherence between the excited molecules, the longer the decay time of the delayed luminescence kinetic curve, and the higher the T value. Therefore, the magnitude of the T value can be used to measure the degree of association between active molecules or tissue order (Xi et al., 2009; Gu, 2013). This study found that the intra-group difference of T was relatively large, indicating that there was a large difference in the correlation between the active molecules within the samples. The only significant difference in T was found between different states of safflower, and there was no significant difference between the other groups, indicating that the applicability of the T value to characterize the quality states of fresh Chinese herbs remains to be investigated.

Alkaloids are the main pharmacodynamic substances in motherwort and have long been its quality control indicators. Leonurine hydrochloride is a special alkaloid in motherwort. Therefore, it can be used as a quality marker for qualitative identification and content determination (Xiong and Peng, 2016). It also has the pharmacological effects of diuresis, anti-platelet aggregation, anti-oxidation, inhibition of creatine kinase activity, and inhibition of the vascular smooth muscle contractile response to vasoconstrictors (Chi et al., 2022; Shen et al., 2023). The results showed that the level of leonurine hydrochloride in the control group was significantly and much higher than the standard level, that in the non-fertilization group was just at the standard level, and that in the drought group was far below the content standard. This showed that there was a significant difference in the content of leonurine hydrochloride in different groups, indicating that its pharmacological action and clinical efficacy would be different, meaning there were differences in the quality of its medicinal properties. These results had the significant correlation with the biophoton detection results. In addition, this study also found that during the growth of motherwort, the content of leonurine hydrochloride first increased and then slightly decreased, which was consistent with literature reports (Zhong, 2015; Qiao, 2019). These studies also showed that the content of alkaloids reached the maximum in the tender motherwort period, then gradually decreased after entering the full leaf period, and rapidly decreased in the fruit period; and that the content in leaves was the highest, followed by flowers, and was the lowest in stems. Therefore, it is recommended to harvest motherwort from the full leaf period to the early flowering period, when the quality of motherwort in this period is relatively high.

It has been reported that quinone chalcone glycosides and other flavonoids are the main active ingredients in safflower (Lei et al., 2020; Guo et al., 2022). Among them, quinone chalcone glycosides are the main active ingredients in safflower (Liu et al., 2017). Currently, HSYA and kaempferol are commonly used as quality control indicators of safflower. HSYA is the most effective water-soluble component of safflower with pharmacological effects, including anticoagulant, antioxidant, anti-inflammatory, anti-myocardial ischemia, and neuroprotective activities (Kong et al., 2022; Wang et al., 2022). It is widely used in the clinical treatment of cardiovascular and cerebrovascular diseases (Shi et al., 2022). Kaempferol is a type of natural flavonoid that exists in large quantities in safflower and has anti-inflammatory, antioxidant, anti-cancer, and other pharmacological effects (Yu et al., 2013; Park et al., 2022). The results showed that there were significant differences in HSYA and kaempferol content of safflower in different growth states, indicating that there were significant differences in the quality, and the results were also significantly correlated with the results of biophoton detection. In addition, the study also found that when the safflower inflorescence changed from yellow to red, around the third day of flowering, the content of active ingredients in safflower was the highest, and therefore, the quality was also relatively high at that time. This indicated that the third day of flowering was the best harvesting time for safflower and was consistent with research results reported in the literature (Hu et al., 2021). This further confirmed that the method of characterizing the quality states of Chinese herbs by the biophoton method is feasible.

It has been reported in the relevant literature that SPE can be used as an effective indicator to identify the quality of fresh Chinese herbs at different growth stages and of different varieties. Furthermore, it can reflect the difference in the content of active ingredients between different growth stages and varieties, which is consistent with the results obtained in this study (Zhao et al., 2017). It has also been reported in the literature that there are differences in the biophoton characteristics of fresh Chinese herbs in different growth stages and different growth states (Zhao, 2014), and it has been proposed that the biophoton characteristics of Chinese herbs can be used to identify the quality of Chinese herbs. The results of this study also showed that there was indeed a significant correlation between the biophoton characteristics and the quality states of fresh Chinese herbs. There was also a significant correlation between the content of active ingredients and pigments and the biophoton characteristics. This confirmed the feasibility of using the biophoton characteristics to evaluate of the quality states of fresh Chinese herbs.

## 5 Conclusion

The quality of Chinese herbs is the basis for the stability and safety in clinical use. There is an urgent need for a method to characterize the quality of fresh Chinese herbs during the growth process, which is in line with the holistic concept of TCM. This paper introduces a new method (the biophoton method) to characterize the quality states of fresh Chinese herbs. We measured the biophoton characteristics, active ingredient content, and pigment content of the motherwort and safflower, and performed the t-test and correlation analysis on these results to obtain the potential relationship between the biophoton characteristics and the quality states of fresh Chinese herbs. Our results show that the biophoton characteristics of fresh Chinese herbs can be correlated with the quality, and their CPS in the steady state and delayed luminescence initial intensity I_0_ can be considered as the characteristic parameters for the quality of fresh Chinese herbs. And the measurement of biophoton characteristics in the growth process is a novel and promising method for the quality evaluation of fresh Chinese herbs.

## Data Availability

The raw data supporting the conclusion of this article will be made available by the authors, without undue reservation.
