# Corrigendum: A biophoton method for identifying the quality states of fresh Chinese herbs

**DOI:** 10.3389/fphar.2023.1195859

**Published:** 2023-04-19

**Authors:** Baorui Cao, Zhiying Wang, Jiayi Zhang, Jialei Fu, Zhongwen Zhang, Jinxin Du, Tingting Deng, Jingxiang Pang, Meina Yang, Jinxiang Han

**Affiliations:** ^1^ Biomedical Sciences College and Shandong Medicinal Biotechnology Centre, First Affiliated Hospital of Shandong First Medical University, Shandong First Medical University and Shandong Academy of Medical Sciences, Jinan, China; ^2^ NHC Key Laboratory of Biotechnology Drugs, Shandong Academy of Medical Sciences, Jinan, China; ^3^ Shandong Academy of Chinese Medicine, Jinan, China; ^4^ Department of Endocrinology, The First Affiliated Hospital of Shandong First Medical University, Jinan, China; ^5^ Shandong University of Traditional Chinese Medicine, Jinan, China

**Keywords:** biophoton, quality state, fresh Chinese herb, motherwort, safflower, UPLC, characteristic parameter

In the published article, the **reference** details for “Qiao, 2019”, “Zhao, 2014” and “Zhong, 2015” were incorrectly written. The correct references are “Qiao, J. J. (2019) Study on the quality evaluation of *Leonurus heterophyllus* L. [master’s thesis]. [Nanjing (Jiangsu)]: Nanjing University Of Chinese Medicine”, “Zhao, X. L. (2014) Preliminary study on the characteristics of biophoton in the growth process of traditional Chinese medicine. [master’s thesis]. [Jinan (Shandong)]: University of Jinan”, and “Zhong, N. Y. (2015) Study on the quality control of *Leonurus japonicus* Houtt. liquid extract and its original medicinal materials. [master’s thesis]. [Hangzhou (Zhejiang)]: Zhejiang Chinese Medical University.”

There was also an error in **Equation 1**. This equation previously stated “CPS = (N − n) × 10” whereas the correct equation is “CPS = N − n.”

The authors apologize for these error and state that they do not change the scientific conclusions of the article in any way. The original article has been updated.

